# Screening Intention Prediction of Colorectal Cancer among Urban Chinese Based on the Protection Motivation Theory

**DOI:** 10.3390/ijerph19074203

**Published:** 2022-04-01

**Authors:** Wenshuang Wei, Miao Zhang, Dan Zuo, Qinmei Li, Min Zhang, Xinguang Chen, Bin Yu, Qing Liu

**Affiliations:** 1 School of Public Health, Wuhan University, Wuhan 430071, China; 2020283050097@whu.edu.cn (W.W.); zhangmiao@whu.edu.cn (M.Z.); 00300654@whu.edu.cn (D.Z.); binyu1029@outlook.com (B.Y.); 2 Wuhan Center for Disease Control and Prevention, Wuhan 430015, China; qinmei.li@whu.edu.cn; 3 Institute of Cancer Prevention and Control, Wuhan 430079, China; hbskaxh@163.com; 4 Department of Epidemiology, University of Florida, Gainesville, FL 32610, USA; jimax.chen@ufl.edu

**Keywords:** colorectal cancer, protection motivation theory, knowledge, screening intention, urban Chinese

## Abstract

Colorectal cancer poses a serious threat worldwide. Although early screening has been proved to be the most effective way to prevent and control colorectal cancer, the current situation of colorectal cancer screening remains not optimistic. The aim of this article is to apply the protection motivation theory (PMT) to examine the influencing factors on screening intention of colorectal cancer (CRC). This cross-sectional survey was launched in five communities in Wuhan, China. All the eligible urban Chinese were recruited and interviewed using paper-and-pencil questionnaires. The intention of colorectal cancer screening (CRCS) was measured using six PMT subconstructs, including perceived risk, perceived severity, fear arousal, response efficacy, response cost, and self-efficacy. Data on sociodemographic variables and knowledge of CRC were also collected. The structural equation modeling (SEM) method was used for data analysis. Among all the 569 respondents, 83.66% expressed willingness to participate in CRCS. Data of the research fit the proposed SEM model well (Chi-square/df = 2.04, GFI = 0.93, AGFI = 0.91, CFI = 0.91, IFI = 0.91, RMSEA = 0.04). Two subconstructs of PMT (response efficacy and self-efficacy) and CRC knowledge were directly and positively associated with screening intention. Age, social status, medical history, physical activity, and CRC knowledge were indirectly related to the screening intention through at least one of the two PMT subconstructs (response efficacy and self-efficacy). The findings of this study suggest the significance of enhancing response efficacy and self-efficacy in motivating urban Chinese adults to participate in CRC screening. Knowledge of CRC is significantly associated with screening intention. This study can provide useful information for the formulation and improvement of colorectal cancer screening strategies and plans.

## 1. Introduction

In 2021, the World Health Organization released new data: Cancer is a leading cause of death worldwide and is expected to remain a major public health problem in the coming years. And colorectal cancer (CRC) is a major cause of cancer burden among both women and men, ranking third in morbidity and second in mortality around the world, causing 1.93 million new cases and almost 916 thousand deaths [1]. Studies from China have shown that the number of CRC has been increasing steadily over the past three decades, from 104.3 thousand cases in 1990 to 555 thousand cases in 2020 and from 81.1 thousand deaths in 1990 to 286 thousand deaths in 2020 [2,3]. Obviously, colorectal cancer is one of the biggest threats to human health, leading to huge social and economic burdens accompanied with a decline in quality of life.

Previous studies showed that CRC morbidity and mortality could be reduced via effective screening and timely treatment [4,5]. Although a range of clinical guidelines have been promoted to achieve prompt screening behaviors for average-risk populations, the uptake in CRC screening remains suboptimal in many countries [6]. The CRC screening rates in the UK and France were 52.0% and 34.3%, respectively [7], while the CRC screening uptake was much lower (27.78%) in Shanghai, China [8]. More seriously, a survey based on the urban cancer early diagnosis and treatment project showed that the screening compliance rate of colorectal cancer was 18.23% among the high-risk population in Hubei Province from 2018 to 2019 [9].

The Health Belief Model (HBM) provides an intuitive conceptual framework to explain preventive practices and has been largely applied to colorectal cancer screening (CRCS) [8,10]. However, the HBM is limited in assessing the deficient effects of severity and vulnerability. Another limitation of the HBM is that this theory is inadequate to offer a clear mechanism through which the HBM components are converted into actual behaviors [11]. Therefore, a more comprehensive and specified model is needed to fully understand preventive behaviors and satisfy greater experimental verification.

Protection motivation theory (PMT) is a comprehensive and systematic model, formulated and revised by Rogers and his colleagues [12]. According to PMT, protection motivation, indexed by behavioral intention, was a latent and reasonable predictor of health behaviors [13]. PMT consists of two different but closely connected cognitive processes: threat appraisal and coping appraisal. And these two processes are sequential; before taking preventive behaviors (coping appraisal) into account, individuals must first recognize and perceive the threat applied to them [12].

Threat appraisal process refers to residents’ perception of maladaptive behaviors or diseases, which contains three subconstructs: risk perception, severity perception, and fear arousal. Risk perception refers to one’s perception of the possibility of illness or adverse health behaviors; it is regarded as the basis of health behavior theories and goals for behavior intervention [14,15]. Severity perception refers to a person’s subjective judgement about the negative consequences induced by diseases or maladaptive behaviors [16]. Fear arousal refers to worries and concerns about being affected by a disease. It is well recognized that risk perception and severity perception may prompt respondents to adopt adaptive behaviors, while fear arousal may hinder the motivation of performing the response [17].

Coping appraisal process in PMT evaluates one individual’s capability to respond to and avert all the threatened danger, including response efficacy, response cost, and self-efficacy. Response efficacy evaluates the belief that one can avert the perceived threat/risk for cancer by performing prevention behaviors [18]. Response cost refers to all possible costs from adopting preventive measures or behaviors, including physical, psychological, and financial costs [19]. Self-efficacy refers to an individual’s expected capability in successfully performing the adaptive response. Evidence from previous studies demonstrated that self-efficacy is an important component of various health behavior theories, influencing motivational, affective, and cognitive processes [20].

Holding that whether an individual produces healthy behavior is influenced by both external environmental factors and intrapersonal factors, PMT has been a common tool to explore motivation and behaviors of individuals in health-related fields and is widely used to predict screening outcomes in diverse tumors, including skin cancer and cervical cancer [21,22].

In the past two decades, the number of colorectal cancer patients has been increasing rapidly, especially in urban areas. As for the poor screening compliance rates, previous literature revealed a lot of barriers, including religion, lack of knowledge and awareness, misunderstanding about the screening tests, etc. [19,23]. However, it remains unclear how sociodemographic factors exert effects on screening intention based on PMT among the asymptomatic urban population. Thus, it is of great significance to carry out a survey among urban Chinese to investigate the CRC intention under the guidance of PMT.

In this study, we aimed to: (1) assess knowledge of CRC and its screening among urban Chinese; (2) identify the possible influencing factors on CRC screening intention of urban Chinese; and (3) investigate the relationship between PMT and colorectal cancer screening intention. This study can give powerful PMT-based evidence to the local government and Health Committee for the development and improvement of colorectal cancer screening strategies among urban Chinese.

## 2. Materials and Methods

### 2.1. Study Design

This cross-sectional study was conducted within the framework of a cancer screening program in urban China (CanSPUC). As a national cancer screening program, CanSPUC has been in progress in urban China since it was first launched in 2012. The objective of CanSPUC is to screen five main types of cancer: lung cancer free, upper gastrointestinal cancer, breast cancer, colorectal cancer, and liver cancer. Detailed information regarding the methodology of CanSPUC has been described in previous studies [24,25].

### 2.2. Study Participants

Briefly, urban residents aged 40 to 74 first received epidemiological questionnaires and high-risk assessments of the five types of cancer. High-risk assessment was based on the Harvard Risk Index and was appropriately adjusted to the characteristics of Chinese [24,26]. Then individuals at high risk for those five types of cancer were screened free of charge. This study consisted of a survey on colorectal cancer screening intention as well as a screening of a high-risk population at baseline. Participants were recruited from five selected communities via convenience sampling method. WeChat, a widely used social application in China, was employed to call on local residents to participate in this survey. Specifically, the staff of the Community Health Center created separated WeChat groups, briefly introduced the purpose of CanSPUC and this study, and encouraged the groups to participate in the study. Thus, eligible participates from the five selected communities were identified and recruited following the Institutional Review Board (IRB) approved protocol.

The inclusion criteria for participation were: (a) 40–74 years old, (b) no clinical diagnosis of CRC, including precancerous lesions, (c) willing to participate in the study and having good understanding power and ability of expression, and (d) no severe organ dysfunction. Residents aged 40–74 years old were selected because of the high CRC risk during this age range [27,28]. Those residents who were diagnosed with CRC were excluded. A total of 598 questionnaires were gathered. Among these questionnaires, 29 with missing data on key demographic variables and CRC knowledge were excluded, yielding a final sample of 569.

### 2.3. Data Collection

The data collection began in September 2020 and lasted for a month via in-person interviews, utilizing paper-and-pencil questionnaires. The questionnaire for data collection was pilot-tested to ensure feasibility and acceptability. Questionnaires were composed of demographic and other factors, knowledge of CRC, and PMT-based scale of CRC. Demographic and other factors were described in detail in the next part, and items about knowledge of CRC and the PMT-based scale of CRC were shown in Appendix A. It took 15–20 min for a typical respondent to complete the survey. Data collectors were second-year public health graduate students from Wuhan University. They received a three-day training session before participating in the data collection. The trained data collectors went to the Community Health Service Centers of the five selected communities to implement the data collection. Completed questionnaires were checked for completeness and accuracy, and double-entry and routine logic checks were performed to detect errors. Disagreements during the data entry were settled by reviewing the original questionnaire.

### 2.4. Variables and Measurements

#### 2.4.1. Intention for CRC Screening

Intention for CRC screening was measured based on participants responses to the following question: “Are you willing to take up CRC screening in the future?” A 5-point Likert-scale was used, with 1 = “very much unwilling” and 5 = “very much willing.” This variable was also dichotomized for analysis: Participants were coded as having no intention if they responded 3 (neutral) or less; otherwise, they were coded as having intention.

#### 2.4.2. Knowledge of CRC

With reference to the previous study [24,29], the CRC Knowledge Scale consisting of 21 items with three subconstructs was developed. (1) the knowledge of risk factors contained 9 items, and a typical item was: “Do you believe the following items are risk factors of CRC or not?” (2) The knowledge of symptoms contained 8 items, and a typical item was: “To your knowledge, which of the following are CRC symptoms?” (3) The screening knowledge contained 4 items, and a typical item was: “Screening is needed for people with bloody stool or diarrhea alternating with constipation.” Participants received one score for a correct answer, with higher scores indicating more knowledge.

#### 2.4.3. PMT-Based Scale of CRC

Combined with the previous research [30], which was a pilot study and expert consultation method, we designed a PMT-based Scale of CRC and revised it extensively to ensure good reliability and validity. Reliability and validity tests were conducted in the previous article [31]. In detail, the Cronbach’s α for the overall scale was 0.763 and ranged from 0.584 to 0.771 for each dimension. The discrimination validity of the scale was examined via correlation coefficients: The correlation coefficients between items within the six dimensions ranged from 0.089 to 0.534, and the correlation coefficients between the dimensions and the total scale ranged from 0.165 to 0.586. The indicators of confirmatory factor analysis were better than the standard values, indicating a good construction validity of the scale.

The PMT-based scale of CRC is composed of two PMT pathways: the threat appraisal and the coping appraisal. The threat appraisal consisted of 3 subconstructs: risk perception (2 items), severity perception (2 items), and fear arousal (2 items). The risk perception referred to one’s assessment of the likelihood of suffering from CRC (Cronbach alpha = 0.584); the severity perception referred to fear of the consequences from CRC (Cronbach alpha = 0.722); and the fear arousal referred to the assessment of worry of being affected by CRC (Cronbach alpha = 0.614). All items were assessed using a 5-point Likert-scale from 1 = “absolutely not” to 5 = absolutely yes.

The coping appraisal consisted of another 3 subconstructs: response efficacy (3 items), response cost (3 items), and self-efficacy (4 items). Response efficacy referred to the judgement about the role of planned colorectal cancer screening in its early detection and the preferable following treatment (Cronbach alpha = 0.737). Response cost referred to the likely economic or social costs of curing or improving CRC (Cronbach alpha = 0.712). Self-efficacy indicated whether participants have enough confidence to adopt regular CRC screening (Cronbach alpha = 0.771). All items were assessed with a 5-point Likert-scale from “1 = strongly disagree” to “5 = strongly agree.”

#### 2.4.4. Demographic and Other Factors

Demographic variables were: age (in years), occupation (administrative/technician, trader/service staff, peasant, housework/no work), education (primary or below, secondary, college or above), social status (created based on occupation and education), regular physical activity (yes/no), lower digestive tract lesions (yes/no), family history of cancer (yes/no). Medical history was formed by the combination of lower digestive tract lesions and family history of cancer. Regular physical activity referred to more than 3 times a week for more than 30 min each time.

### 2.5. Statistical Analysis

Descriptive statistics (i.e., mean, standard deviation (SD), proportion, and rate) were used to describe the study sample and the distribution of key variables. The chi-square test and Student’s t-test were performed to compare differences in factors between respondents with and without screening intention. Structural equation modeling (SEM) analysis was used to examine PMT-based models linking influential factors with screening intention. Data-model fit was assessed using multiple criteria, including GFI (>0.90), AGFI (>0.90), CFI (>0.90), IFI (>0.90), RMSEA (<0.08), and chi-square/df ratio (<3.0). *p* < 0.05 (two-sided) was used for statistical inference. Statistical analyses were conducted using IBM Statistical Package for the Social Sciences (SPSS) version 25.0 and Analysis of Moment Structures (Amos) version 21.0 for Windows (Chicago, IL, USA).

## 3. Results

### 3.1. Sample Characteristics

Table 1 summarizes the basic characteristics for the study sample (n = 569). The average age of the total study population was 59.05 (SD = 8.54) years old. The male to female ratio was 1:1.03. Of the total sample, 10.02% were farmers and 86.11% had secondary or more education. Approximately 70% (69.07%) of the total population reported having regular physical activity. Among all the subjects, those with lower digestive tract lesions and a family history of cancer accounted for 12.65% and 38.31%, respectively. 83.66% of all participates expressed willingness to participate in CRCS. There were no significant differences on screening intention among different characteristics groups (*p* > 0.05).

### 3.2. Knowledge of CRC

Table 2 exhibits the knowledge scores of CRC, containing risk factors, symptoms, and screening. The results from the Student’s t-test revealed that people with screening intention had higher scores about CRC knowledge compared with those participates without intention (mean = 14.16, SD = 4.56 vs. Mean = 12.43, SD = 5.67, *p* = 0.001). Moreover, the comparisons of each subscale between the two groups (with and without screening intention) showed that those respondents who had screening intention got higher scores than those without screening intention. The typical items regarding knowledge of CRC risk factors and symptoms were “smoking frequently” (62.18% vs. 44.09%, *p* = 0.001) and “change in bowel habits” (77.10% vs. 59.14%, *p* < 0.001).

### 3.3. Correlation between CRC Screening Intention, PMT Subconstructs, and CRC Knowledge

Table 3 presents the results from Pearson correlation analysis. Screening intention X1 was significantly related to X8, the CRC knowledge score (r = 0.24, *p* < 0.01), and five PMT subconstructs, particularly X7, the subconstruct of self-efficacy (r = 0.52, *p* < 0.01). In addition, CRC knowledge was significantly associated with four of the six PMT subconstructs except risk perception and severity perception. Three subconstructs of PMT (response efficacy X5, response cost X6, and self-efficacy X7) were all significantly associated with subdomains of the CRC knowledge scale (knowledge of CRC risk factors X9, knowledge of CRC symptoms X10, knowledge of CRC screening X11).

### 3.4. Analysis of Structure Equation Model

**Figure 1:**
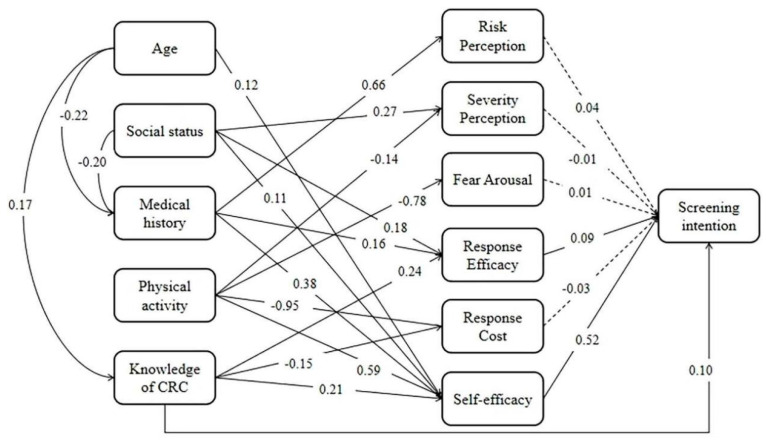
A structure equation modeling factors influencing screening intention. Note: A solid line indicates that the relationship is statistically significant (*p* < 0.05), and a dashed line indicates that the relationship had no statistical significance (*p* > 0.05). The data−model fit index: GFI = 0.93, AGFI = 0.91, CFI = 0.91, IFI = 0.91, RMSEA = 0.04, Chi−square/df = 2.04.

All data fit the proposed structural equation models quite well, with GFI = 0.93, AGFI = 0.91, CFI = 0.91, IFI = 0.91, RMSEA = 0.04, and chi−square/df = 2.04. Results from the modeling are presented in Figure 1. First, of the six PMT constructs, two (self-efficacy and response efficacy) were significantly associated with CRC screening intention. The self-efficacy measures the perceived ability to take part in CRC screening, while the response efficacy measures the perceived effectiveness of screening in CRC prevention and treatment.

All the four external factors (social status, medical history, physical activity, and CRC knowledge) were significantly associated with the PMT subconstruct of self-efficacy, suggesting the role of these factors in promoting CRC screening through self-efficacy. In addition, age was positively associated with self-efficacy and CRC knowledge was directly and positively associated with the intention for CRC screening.

Of the four external factors, three (social status, medical history, and CRC knowledge) were associated with response efficacy, another PMT subconstruct that was significantly associated with CRC screening intention. In other words, these factors can promote screening intention by increasing the perceived effectiveness of screening for CRC prevention and treatment.

## 4. Discussion

In this current study, a multivariate approach-SEM was utilized to explore relationships between colorectal cancer screening intention and the two pathways of PMT (threat appraisal process and coping appraisal process). To our knowledge, this is the first study to assess colorectal cancer screening intention by employing theoretical framework PMT among urban Chinese. The findings of this study enable us to add new evidence to advance colorectal cancer research around the world, there being currently millions of colorectal cancer patients. The findings can strengthen the theoretical basis of the underlying mechanisms regarding urban Chinese screening intention and behaviors. All these data can promote the development of behavioral theory–based programs and increase CRC screening rates.

Results from SEM indicated the good fit of the whole model, which meant that PMT was a good tool to explore the relationships between sociodemographic factors and screening intention. Consistent with the findings of other researchers, not all constructs of PMT were equally strong in predicting health-related behaviors [21,32,33]. Data from this study indicated that the two PMT variables (response efficacy and self-efficacy) had significant associations with CRC screening intention, demonstrating a stronger impact on the perception of coping appraisal than on the perception of threat appraisal [34].

Notably, findings of this research suggested that self-efficacy was the most powerful predictor of the six subconstructs of PMT, similar to research findings of other investigators [32,35,36]. This positive connection showed that those individuals with greater perceptions of self-efficacy tended to overcome more obstacles, and had much more confidence to participate in CRC screening [37,38]. Hence, it is necessary to emphasize self-efficacy when designing and carrying out various health education interventions to further improve screening uptake and mitigate the threat of CRC [39,40].

As indicated by the results of previous surveys and the present study, response efficacy should also be considered in an effective screening program of colorectal cancer, acting as a promoting factor for screening willingness and behaviors [41,42]. Participants perceiving higher response efficacy were more likely to believe in the benefits of CRCS and have stronger intention of CRCS, while low response efficacy was reported to be associated with consistent refusal of screening participation [43,44]. This finding was important for urban Chinese because they lacked adequate education about CRC and enough perception of effectiveness regarding screening [45].

Another noteworthy discovery of this study facilitates effective implementation of PMT-based screening policies and prevention activities. Results from pathway analysis suggested that knowledge of CRC had a positive relationship with the scores of screening intention both in direct and indirect pathways. Likewise, published literature studies revealed that the respondents who got higher scores in knowledge were more likely to participate in CRC screening [46,47]. Those who had deficient knowledge of CRC risk factors, clinical symptoms, and detection would be discouraged from attending screening. So, implementation of educational interventions on the part of participants and staff of the primary healthcare system for removing barriers to CRC knowledge was quite essential, as it has already been proved to be a successful facilitator of CRC screening [48].

Findings from this survey reinforce the importance of focusing on the key social demographic and other factors. Age, physical activity, social status (education and occupation), and medical history (lower digestive tract lesions and family history of cancer) were found to be correlated to the screening intention mediated through at least one of the two PMT subconstructs (response efficacy and self-efficacy). Previous studies have shown that people with a personal history of colorectal disease were more likely to undergo CRC screening, whilst the uptake of screening was lower among older residents and those of lower socioeconomic status [49,50]. Individuals of higher social status and regular physical activity tend to pay more attention to their health and hold a positive attitude to CRCS. Therefore, targeted educational campaigns and screening programs based on PMT should be designed and established, taking the above factors into account.

Although this study found that only two subconstructs of PMT were significantly related to screening intention, it was not meant to imply that those subconstructs with no statistical significance made no contributions. The previous reports found that risk perception and severity perception were influencing factors of CRC screening [51,52]. Moreover, in this model, the total variance of the outcome variable was only partially explained, suggesting that other possible influencing factors were not included and further studies were needed.

Nevertheless, some limitations of this survey need to be noted. Firstly, the generalization of the results from this study should be considered with caution given the possible sampling selection bias, since the study sample was obtained from five communities in one city of central China via convenience sampling and the sample size was small. Secondly, due to the inherent deficiency of cross-sectional design, the causal relationship between screening intention and other factors can’t be warranted. Therefore, multi-center cohort studies with large samples will be needed in the future to produce stronger evidence. Third, in view of possible reporting bias due to time constraints, the question about screening intention could be further refined in future studies, for example, “Do you plan to have a CRC screening in the next three (or five) years?”

## 5. Conclusions

Despite these limitations, this is the first study to assess colorectal cancer screening intention among urban Chinese based on PMT. Moreover, the results of the study offer new data supporting the implementation of effective theory-based cognitive behavioral prevention interventions to enhance willingness to receive colorectal cancer screening. Most importantly, the success of this survey indicates that the PMT developed in the United States could also be used as a good tool to investigate willingness of colorectal cancer screening among urban residents in other developing countries.

## Figures and Tables

**Table 1:** The demographic characteristics of the study population.

Variable	Screening Intention		Total (%)	*p* Value
	Yes	No		
Sample size, N (%)	476 (83.66)	93 (16.34)	569 (100.00)	
Age in years, n (%)				0.275
40–49	78 (16.39)	19 (20.43)	97 (17.05)	
50–59	135 (28.36)	31 (33.33)	166 (29.17)	
60–74	263 (55.25)	43 (46.24)	306 (53.78)	
Mean (SD)	59.33 (8.53)	57.61 (8.54)	59.05 (8.54)	0.730
Social status				
Occupation, n (%)				0.148
Administrative/technician	182 (38.24)	33 (35.48)	215 (37.79)	
Trader/service staff	234 (49.16)	41 (44.09)	275 (48.33)	
Peasant	45 (9.45)	12 (12.90)	57 (10.02)	
Housework/No work	15(3.15)	7 (7.53)	22 (3.87)	
Educational level, n (%)				0.662
Primary or below	65 (13.66)	14 (15.05)	79 (13.88)	
Secondary	266 (55.88)	55 (59.14)	321 (56.41)	
College or above	145 (30.46)	24 (25.81)	169 (29.70)	
Regular physical activity, n (%)				0.126
Yes	335 (70.38)	58 (62.37)	393 (69.07)	
No	141 (29.62)	35 (37.63)	176 (30.93)	
Medical history				
Lower digestive tract lesions, n (%)				0.674
Yes	59 (12.39)	13 (13.98)	72 (12.65)	
No	417 (87.61)	80 (86.02)	497 (87.35)	
Family history of cancer, n (%)				0.280
Yes	187 (39.29)	31 (33.33)	218 (38.31)	
No	289 (60.71)	62 (66.67)	351 (61.69)	

Note: SD was standard deviation.

**Table 2:** Comparisons of knowledge of CRC among urban residents with and without colorectal cancer screening intention.

Total Scale/Single Item	Screening Intention		*p* Value
	Yes	No	
Total scale score, mean (SD)	14.16 (4.56)	12.43 (5.67)	0.001
Knowledge of CRC risk factors			
Subscale score, mean (SD)	5.88 (2.54)	5.03 (3.08)	<0.001
Older age, n (%)	219 (46.01)	32 (34.41)	0.039
Family cancer history, n (%)	286 (60.08)	42 (45.16)	0.008
Low vegetables fruits intake, n (%)	341 (71.64)	59 (63.44)	0.114
Frequent high-fat food intake, n (%)	342 (71.85)	59 (63.44)	0.104
Frequent meat intake, n (%)	292 (61.34)	54 (58.06)	0.553
Obesity, n (%)	266 (55.88)	47 (50.54)	0.343
Lack of physical activity, n (%)	350 (73.53)	62 (66.67)	0.176
Eating fried food frequently, n (%)	409 (85.92)	72 (77.42)	0.038
Smoking frequently, n (%)	296 (62.18)	41 (44.09)	0.001
Knowledge of CRC symptoms			
Subscale score, mean (SD)	5.30 (2.22)	4.52 (2.66)	0.010
Blood in stool, n (%)	421 (88.45)	71 (76.34)	0.002
Mucus in stool, n (%)	337 (70.80)	57 (61.29)	0.069
Change in bowel habits, n (%)	367 (77.10)	55 (59.14)	<0.001
Diarrhea or constipation, n (%)	357 (75.00)	64 (68.82)	0.214
Abdominal and anal pain, n (%)	330 (69.33)	50 (53.76)	0.004
Vomit, n (%)	172 (36.13)	29 (31.18)	0.361
Anemia, n (%)	179 (37.61)	32 (34.41)	0.559
Weight loss, n (%)	360 (75.63)	62 (66.67)	0.071
Knowledge of CRC screening			
Subscale score, mean (SD)	2.98 (0.86)	2.88 (0.91)	0.057
No need to re-screen if normal from a previous screen, n (%)	322 (67.65)	57 (61.29)	0.234
Screening is needed for people with bloody stool or diarrhea alternating with constipation, n (%)	444 (93.28)	87 (93.55)	0.924
People with family history of colorectal polyps or CRC must screen, n (%)	435 (91.39)	76 (81.72)	0.005
CRC screening tests include fecal occult blood tests and colonoscopy, n (%)	385 (80.88)	69 (74.19)	0.142

Note: The comparison of mean scores between the two groups was implemented via Student’s t-test, and the comparison of each item between the two groups was via chi-square test.

**Table 3:** Inter-correlations for the screening intention and some variables.

Variables	X1	X2	X3	X4	X5	X6	X7	X8	X9	X10	X11
X1	1.00	0.16 **	0.01	−0.23 **	0.27 **	−0.33 **	0.52 **	0.24 **	0.22 **	0.20 **	0.11 **
X2		1.00	0.06	0.08	0.05	0.01	0.17 **	0.07	0.07	0.10 *	−0.07
X3			1.00	0.14 **	0.16 **	0.05	0.04	0.04	0.01	0.05	0.07
X4				1.00	−0.18 **	0.50 **	−0.33 **	−0.09 *	−0.09 *	−0.08	−0.03
X5					1.00	−0.22 **	0.33 **	0.17 **	0.14 **	0.14 **	0.16 **
X6						1.00	−0.49 **	−0.16 **	−0.12 **	−0.14 **	−0.15 **
X7							1.00	0.20 **	0.17 **	0.19 **	0.10 *
X8								1.00	0.89 **	0.88 **	0.46 **
X9									1.00	0.61 **	0.24 **
X10										1.00	0.32 **
X11											1.00

Note: X1: screening intention; X2: risk perception; X3: severity perception; X4: fear arousal; X5: response efficacy; X6: response cost; X7: self-efficacy; X8: total CRC knowledge score; X9: knowledge of CRC risk factors; X10: knowledge of CRC symptoms; X11: knowledge of CRC screening. X2 to X7 were the six subconstructs of PMT. ** *p* < 0.01, * *p* < 0.05.

## Data Availability

The data presented in this study are openly available contacting corresponding Author.

## Appendix A

**Table A1:** Items of the 21-item for CRC knowledge questionnaire.

Items by Subconstructs
Knowledge of CRC risk factors
Q1. Older age.
Q2. Family cancer history.
Q3. Low vegetables fruits intake
Q4. Frequent high-fat food intake.
Q5. Frequent meat intake.
Q6. Obesity.
Q7. Lack of physical activity.
Q8. Eating fried food frequently.
Q9. Smoking frequently.
Knowledge of CRC symptoms
Q10. Blood in stool.
Q11. Mucus in stool.
Q12. Change in bowel habits.
Q13. Diarrhea or constipation.
Q14. Abdominal and anal pain.
Q15. Vomit.
Q16. Anemia.
Q17. Weight loss.
Knowledge of CRC screening
Q18. No need to re-screen if normal from a previous screen.
Q19. Screening is needed for people with bloody stool or diarrhea alternating with constipation
Q20. People with family history of colorectal polyps or CRC must screen.
Q21. CRC screening tests include fecal occult blood tests and colonoscopy.

**Table A2:** Items of the 16-item colorectal cancer PMT scale.

Items by Subconstructs
Risk perception
Q1. I am more prone to colorectal cancer than others.
Q2. I have been reminded by someone to be cautious of getting colorectal cancer.
Severity perception
Q3. Probability of death is high after one is diagnosed with colorectal cancer.
Q4. A person who gets colorectal cancer is hard to be cured.
Fear arousal
Q5. I don’t want to undergo colonoscopy for fear of getting colorectal cancer.
Q6. I’m afraid to talk to someone else about colorectal cancer.
Response efficacy
Q7. Colorectal cancer can be examined early via doing colonoscopy.
Q8. Through colonoscopy, I can find out if it is colorectal cancer.
Q9. If one would like to cure colorectal cancer, it can’t be done without colonoscopy.
Response cost
Q10. Going to a hospital for colonoscopy wastes a lot of time.
Q11. It takes a long way to a hospital for colonoscopy.
Q12. I felt very embarrassed to go to the hospital for colonoscopy.
Self-efficacy
Q13. Colonoscopy is easy to accept for me.
Q14. I have sufficient time to go to a hospital for colonoscopy.
Q15. Even though other people say colonoscopy is not necessary, I will go for it myself.
Q16. Even though colonoscopy costs me money, I will do it.
